# Exploring the Antimicrobial Properties of 99 Natural Flavour and Fragrance Raw Materials against Pathogenic Bacteria: A Comparative Study with Antibiotics

**DOI:** 10.3390/plants12213777

**Published:** 2023-11-06

**Authors:** Zuzanna Bacińska, Kinga Baberowska, Alicja Karolina Surowiak, Lucyna Balcerzak, Daniel Jan Strub

**Affiliations:** 1 Department of Chemical Biology and Bioimaging, Faculty of Chemistry, Wrocław University of Science and Technology, Wyb. Wyspiańskiego 27, 50-370 Wrocław, Poland; 2 Department of Engineering and Technology of Chemical Processes, Faculty of Chemistry, Wrocław University of Science and Technology, Wyb. Wyspiańskiego 27, 50-370 Wrocław, Poland; 3 Department of Analytical Chemistry and Chemical Metallurgy, Faculty of Chemistry, Wrocław University of Science and Technology, Wyb. Wyspiańskiego 27, 50-370 Wrocław, Poland

**Keywords:** Gram-positive bacteria, Gram-negative bacteria, natural fragrance raw materials, essential oils, MIC

## Abstract

Currently, one of the most serious global problems is the increasing incidence of infectious diseases. This is closely related to the increase in antibiotic use, which has resulted in the development of multidrug resistance in microorganisms. Another problem is the numerous microbiological contaminations of cosmetic products, which can lead to dangerous bacterial infections in humans. Natural fragrance raw materials exhibit a wide spectrum of biological properties, including antimicrobial properties. Despite their prevalence and availability on the commercial market, there is little research into their effects on multidrug-resistant microorganisms. This study examines the inhibitory effect of natural substances on Gram-positive and Gram-negative bacteria. For this purpose, screening and appropriate assays were carried out to determine the minimum inhibitory concentration (MIC) value of individual substances, using the alamarBlue^TM^ reagent. The lowest MIC values were observed for *Staphylococcus aureus* (black seed (*Nigella sativa*) expressed oil, MIC = 25 µg/mL), *Kocuria rhizophila* (fir balsam absolute, MIC = 12.5 µg/mL), and *Pseudomonas putida* (cubeb oil and fir balsam absolute, MIC = 12.5 µg/mL). The most resistant Gram-negative species was *Enterobacter gergoviae*, while *Staphylococcus epidermidis* was the most resistant Gram-positive species.

## 1. Introduction

Multiple drug resistance (MDR) is defined as the resistance of a microorganism to at least one antimicrobial drug in three or more categories [1]. Currently, it poses a significant obstacle to the treatment of bacterial and fungal infections in patients due to the limited possibility of selecting an effective and selective antibiotic therapy [2]. The presence of resistant microorganisms in the hospital environment is a very serious problem that makes it difficult to perform surgeries, among other things. The main cause of resistance is the excessive and inappropriate use of antibiotics, as well as their widespread use in industries such as agriculture, food, and veterinary medicine in rapidly developing countries [3]. Therefore, it is essential to find natural substances that can inhibit the growth of bacteria. It is important to note that bacteria of the same species are not always resistant or sensitive to a given antimicrobial compound in the same way. Resistance and susceptibility are determined by the minimum inhibitory concentration (MIC) of the biocidal compound that inhibits the growth of the microorganism.

**Figure 1:**
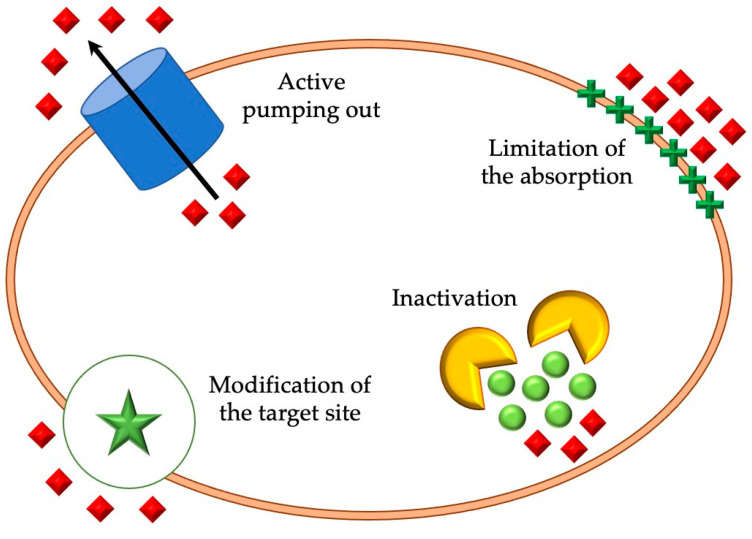
The main mechanisms of resistance in bacteria. Own elaboration.

Microorganisms have developed specific mechanisms to survive in the presence of toxic compounds due to their adaptation to various environmental conditions. Bacteria use mechanisms that can be classified into four categories: absorption of limitation of substances, modification of the target site, inactivation, and active pumping out of the cell interior [4]. The main mechanisms of resistance in bacteria is shown in Figure 1. Red squares indicate substances toxic to bacteria.

So far, antibiotics and synthetic chemicals with antibacterial activity have been the most effective and widely used tools against pathogens. However, due to increasing multidrug resistance, other solutions should be sought. Taking into account the growing global market of natural fragrance raw materials, essential oils, absolutes, balsams, and concretes show great potential in this area.

Essential oils (EOs) are secondary metabolites that have a characteristic scent. Their secretion aims to protect against parasites and predators, limit the growth of competing plants, and prevent sprouting in the winter. In addition, EOs play an important ecological role in ecosystems, where they act as attractants and repellents. They are soluble in alcohols and ethers, but insoluble in water [5]. Their lipophilic nature allows them to penetrate the cell wall and the cytoplasmic membrane of bacteria, causing their integrity and structure. The presence of various chemical compounds in EOs can reduce the membrane potential, interfere with the proton pump activity, coagulate the cytoplasm, and degrade structures such as lipids or proteins [6]. Consequently, these activities lead to the leakage of cellular organelles into the environment and the lysis of the bacterial cell [6]. EOs exhibit a wide spectrum of biological properties, including antimicrobial, antiviral, antifungal, antiparasitic, antioxidant, and insecticidal [7]. The properties of the natural material depend on the main bioactive components. Numerous studies have confirmed the antimicrobial activity of natural fragrance raw materials [2,5,7,8,9,10,11,12]. Due to their antimicrobial properties, they can be used in the fight against pathogens in the cosmetics, pharmaceutical, and food industries.

The purpose of this study was to determine the inhibitory effect of selected natural fragrances, which were essential oils, balsams, concretes, and absolutes, on selected species of microorganisms that are considered pathogens capable of developing antibiotic resistance and contributing to the development of diseases. These bacterial strains were selected due to their significant pathogenic capacity and high risk of product contamination in various areas. The results of this study may help find natural alternatives to antiseptics and antibiotics that will be equally effective against antibiotic-resistant microorganisms. In this study, we used Gram-negative bacteria from the genera *Pluralibacter*, *Klebsiella*, *Pseudomonas*, and *Burkholderia*, and Gram-positive bacteria from the genera *Staphylococcus*, *Kocuria*, and *Cutibacterium*. These bacteria are natural members of the skin microbiota and are not a threat in the case of healthy skin tissue. However, many studies have shown that these species are closely related to skin diseases, including atopic dermatitis or acne [13,14,15]. Numerous other parts of the body can be colonised by pathogens, including the axillae, groin, and gastrointestinal tract. Colonisation provides a reservoir from which bacteria can be introduced into the bloodstream when the host defence is disrupted, whether by shaving, aspiration, or surgery [16]. In the case of *S. aureus*, its presence can cause, for example, pneumonia, respiratory tract infections, endocarditis, osteomyelitis, conjunctivitis, and other diseases [17]. Furthermore, the presence of bacteria in damaged skin tissue leads to the development of wounds, bacterial infections, and difficulties in healing [12]. One of the most extreme developments in skin and soft tissue infections is necrotising fasciitis and necrotising soft tissue infections caused by the Streptococcus A group and methicillin-resistant *S. aureus* (MRSA) [18]. In our work, we focus on the use of essential oils and other fragrances due to their availability, simplicity of use, and biological properties. Due to their characteristic fragrances, they are mainly used in aromatherapy, where they have a relaxing function, improving emotional and physical health by penetrating subcutaneous tissues [19]. Therefore, we focused on the use of natural fragrances as growth inhibitors of selected bacterial species.

## 2. Results

### 2.1. Screening Assays

**Figure 2:**
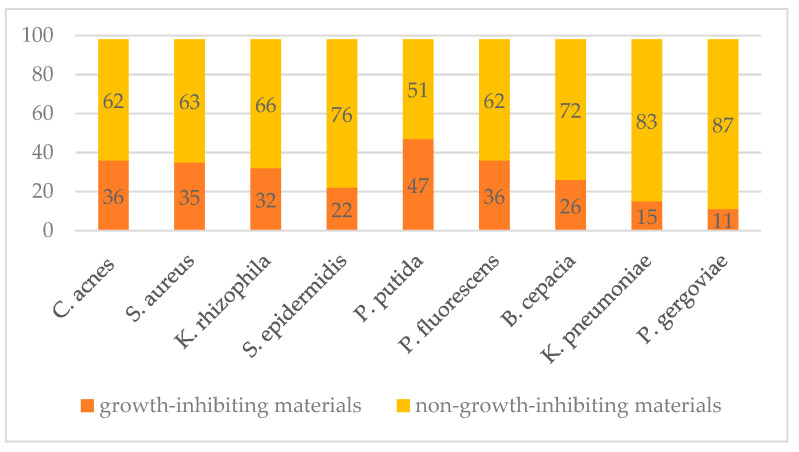
Summary of the amount of inhibitory and non-inhibitory materials against all tested bacterial species.

The first part of the study was devoted to screening assays for each microbial species to identify raw materials that showed an inhibitory effect on bacterial growth. The assays were carried out with the use of alamarBlue^TM^ reagent. The number of natural fragrances that showed an inhibitory effect (at a concentration of 200 µg/mL) on the growth of the tested bacterial species is as follows: out of 99 natural fragrance raw materials, 47 showed an inhibitory effect on the growth of *Pseudomonas putida*, 36 on *Cutibacterium acnes* and *Pseudomonas fluorescens*, 35 on *Staphylococcus aureus*, 32 on *Kocuria rhizophila*, 26 on *Burkholderia cepacia*, 22 on *Staphylococcus epidermidis*, 15 on *Klebsiella pneumoniae*, and 11 on *Pluralibacter gergoviae* (Figure 2).

### 2.2. Results of the MIC Assay 

#### 2.2.1. MIC Evaluation—Gram-Positive Bacteria

The MIC values for the most active natural fragrance materials against all the evaluated strains are presented in Table 1. Regarding the *S. aureus* strain, the most effective bacteriostatic activity was found for raw materials such as *Nigella sativa* expressed oil (black seed) (MIC = 25 µg/mL) and *Callitris intratropica* essential oil (blue cypress) (MIC = 50 µg/mL). For the *S. epidermidis* strain, the lowest MIC values were observed with natural fragrance raw materials such as *Juniperus communis* CO_2_ extract (juniper berry) (MIC = 25 µg/mL), *Coriandrum sativum* oil (coriander herb), *C. intratropica* essential oil (blue cypress), and *Abies balsamea* absolute (fir balsam) (MIC = 50 µg/mL). Regarding *K. rhizophila*, the fragrances with the lowest MIC values were *A. balsamea* absolute (fir balsam) (MIC = 12.5 µg/mL), *Copaifera officinalis* balsam (copaiba), *J. communis* CO_2_ extract (juniper berry), and *C. sativum* oil (coriander herb) (MIC = 25 µg/mL). The essential oils of *Angelica archangelica* (angelica root), *Piper cubeba* (cubeb), and *A. balsamea* absolute (fir balsam) showed inhibitory effects on *C. acnes* bacteria with a MIC of 25 µg/mL.

Of all the natural fragrance raw materials tested, the best results against Gram-positive species were obtained using *Piper nigrum* oleoresin 40/20 (pepper black), *J. communis* CO_2_ extract (juniper berry), essential oils of *C. sativum* (coriander herb), *C. intratropica* essential oil (blue cypress), *Fokienia hodginsii* (siam wood), and *A. balsamea* absolute (fir balsam), as well as *Iris pallida* concentrate (orris root). These substances exhibited significant inhibitory activity against the growth of each bacterial strain.

In particular, *S. epidermidis* was found to be the most resistant to the action of natural fragrances, as evidenced by its inhibition by only 22 fragrance materials, while *C. acnes* was the least resistant, with inhibition by 36 fragrance materials.

#### 2.2.2. MIC Evaluation—Gram-Negative Bacteria

The results obtained for Gram-negative bacteria are as follows: for *K. aerogenes*, the most effective substances were *J. communis* CO_2_ extract (juniper berry) (MIC = 25 µg/mL), *C. sativum* oil (coriander herb) (MIC = 25 µg/mL), *A. balsamea* absolute (fir) (MIC = 25 µg/mL), and *C. intratropica* essential oil (blue cypress) (MIC = 50 µg/mL). For *K. pneumoniae*, the most effective substance was *C. sativum* oil (coriander herb) (MIC = 50 µg/mL), while for *E. gergoviae*, it was *J. communis* CO_2_ extract (juniper berry) (MIC = 50 µg/mL). *C. intratropica* essential oil (blue cypress) was the most effective substance for *B. cepacia* (MIC = 25 µg/mL), whereas *J. communis* CO_2_ extract (juniper berry) and *A. balsamea* absolute (fir) were the most effective substances for *P. fluorescens* (MIC = 50 µg/mL). For *P. putida*, the most effective substances were *Piper cubeba* (cubeb) and *A. balsamea* absolute (fir) (MIC = 12.5 µg/mL). The results summarised in Table S1 indicate that *P. putida* is the least resistant Gram-negative bacteria, as most of the raw materials showed an MIC value of around 400 µg/mL (more information is included in the supplement).

Regarding the number of fragrance materials that inhibit the tested pathogens, *P. gergoviae* is the most resistant species, as only 11 fragrance raw materials inhibited its growth, which is the smallest number among all the tested Gram-negative species. On the other hand, *P. putida* is the least resistant bacteria species, as it was inhibited by 47 fragrance raw materials.

All MIC values obtained for each of the bacterial strains are presented in Table S1. The most effective raw materials for each type of Gram-negative bacteria were essential oils of *C. sativum* and *C. intratropica*, *J. communis* CO_2_ extract, and *A. balsamea* absolute, which had the lowest MIC values.

#### 2.2.3. MIC Evaluation of Antibiotics against Tested Bacteria

MIC values were determined for antibiotics such as gentamicin, ciprofloxacin, ampicillin, and amfotericin B. Gentamicin was tested on *S. aureus*, *K. rhizophila*, *P. fluorescens*, and *P. putida*, species with MIC values of 5 µg/mL, 1 µg/mL, 2 µg/mL, and 0.625 µg/mL, respectively. Ciprofloxacin inhibited the growth of *K. rhizophila* with an MIC = 0.625 µg/mL, *K. pneumoniae* with an MIC = 0.50 µg/mL, *B. cepacia* with an MIC *=* 0.25 µg/mL*, P. fluorescens* with an MIC = 0.008 µg/mL, and *P. putida* and *K. pneumoniae* with an MIC = 0.019 µg/mL. For ampicillin, the MIC values were determined for *P. gergoviae* (MIC = 0.008 µg/mL), *S. epidermidis* (MIC = 0.0625 µg/mL), and *C. acnes* (MIC = 0.25 µg/mL). All results are presented in Table S1.

## 3. Discussion

The antimicrobial activity of essential oils has been confirmed in many studies [5,7,8,9,10,11]. In this study, we evaluated the antibacterial activity of ninety-nine fragrance raw materials against Gram-positive and Gram-negative pathogens. The essential oils of *C. intratropica* and *C. sativum*, *N. sativa* expressed oil, *A. balsamea* absolute, and *J. communis* CO_2_ extract showed the lowest MIC values (12.5–50 µg/mL) for all evaluated species. Previous studies have shown that natural fragrance materials are more active against Gram-positive strains than Gram-negative strains [20,21,22]. This is due to the structure of the cell wall and the natural resistance of Gram-negative bacteria caused by the presence of a double layer of phospholipids and LPS [23]. However, in our study, we did not observe this dependence: the MIC values were the same or very similar for both Gram-positive and Gram-negative bacteria. For example, the MIC values for *J. communis* CO_2_ extract for *S. epidermidis* and *P. gergoviae* were 50 µg/mL, and for *A. balsamea* absolute for *K. rhizophila* and *P. putida*, the MIC values were 12.5 µg/mL.

Most of the studies conducted so far have focused on the Gram-positive microorganisms of the *S. aureus* species, and there is still limited research into the effects of natural fragrance materials on other Gram-negative bacteria besides the *E. coli* species [21,24,25,26,27]. Furthermore, there is a paucity of the literature investigating the effects of natural fragrance materials on bacterial species belonging to the genera *Burkholderia*, *Pseudomonas*, *Klebsiella*, *Cutibacterium*, and *Kocuria.* In existing studies, the MIC values are often given as the zone of inhibition of growth or concentration % (*v*/*v*) [21,24,28,29]. To compare the results obtained with those of other authors, it is crucial to use the same method, culture conditions, specific bacterial strains, and tested fragrance compounds. Therefore, it is challenging to compare the obtained MIC values with other data.

On the basis of the MIC values obtained, several natural fragrance raw materials that exhibited the lowest MIC values were selected for the tested bacterial strains. For the selected raw materials, the main components that occur in their composition are presented in Table 2.

**Figure 3:**
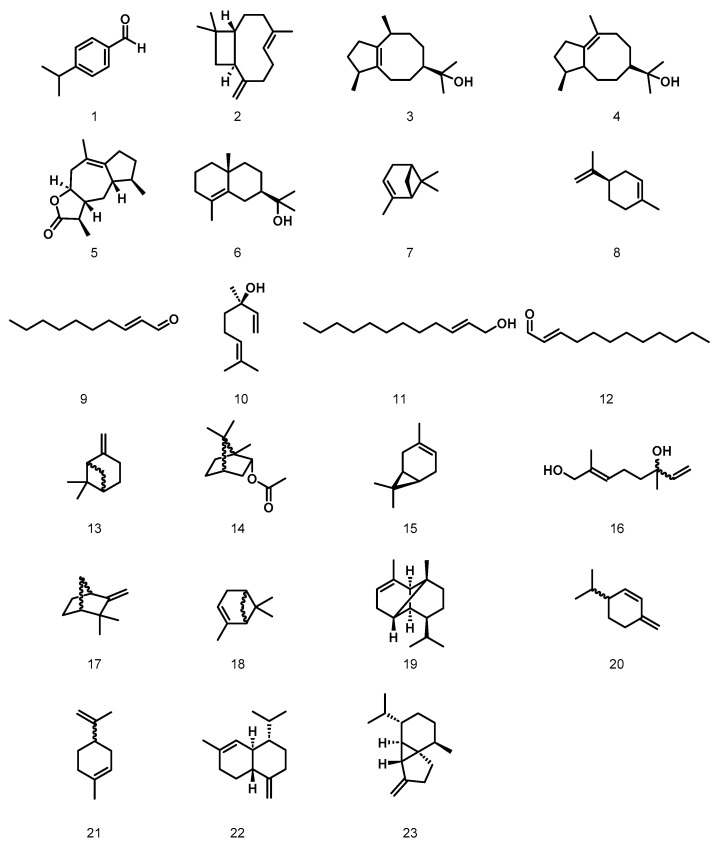
Structures of the main components of natural fragrance raw materials showing the lowest MIC values.

Each of the selected natural raw materials listed in Table 2 has a different composition. Therefore, their antibacterial activity differs from that of the microorganisms tested. The composition and properties of essential oils and extracts are influenced by the time of harvesting the plant, the conditions under which it was grown, light interception, the part of the plant from which the raw fragrance material was extracted, or the manner and conditions under which the extraction process was carried out [40]. The structures of the main components of natural fragrance raw materials showing the lowest MIC values are shown in Figure 3.

In the case of *N. sativa* expressed oil, for which the lowest MIC value was shown for *S. aureus* (MIC = 25 µg/mL), the main compounds found in this raw material are cuminaldehyde **1** and β-caryophyllene **2** [30]. Li et al. showed that cuminaldehyde inhibits the growth of *S. aureus* (ATCC 6538) with an MIC result of 800 µg/mL, confirming its antibacterial properties [41]. Chew Li Moo confirmed the biocidal properties for β-caryophyllene for *Bacillus cereus* (ATCC 14579), but not for *S. aureus* [42]. However, these results make it possible to conclude that *N. sativa* expressed oil containing mainly cuminaldehyde and β-caryophyllene in the volatile fraction has antibacterial properties. 

The essential oil of *C. intratropica,* which showed the highest antimicrobial activity for *P. putida* and *B. cepacia* (MIC = 25 µg/mL), contains mainly guaiol **3**, bulnesol **4**, dihydrocolumellarin **5**, and γ-eudesmol **6** [31,32]. Petard showed that the essential oil of *Bulnesia sarmienti*, which consists mainly of bulnesol and guaiol, exhibits an inhibitory effect on the growth of Gram-positive bacteria [43]. This allows us to conclude that, despite the different natural raw materials tested, the high percentage of bulnesol and guaiol influences the biocidal activity of the fragrance raw material.

In the case of the *J. communis* CO_2_ extract, the lowest MIC values were shown for *K. rhizophila, S. epidermidis,* and *K. aerogenes* (MIC = 25 µg/mL). The main components of the volatile fraction of this raw material are (-)-α-pinene **7** and D-limonene **8** [33,34]. Dhar et al. found no antibacterial activity exhibited by (-)-α-pinene against *E. coli* and *S. aureus* [44]. Silva et al. also found no antibacterial activity for (-)-α-pinene against *S. aureus* [45]. In the case of limonene, Han et al. confirmed its antibacterial activity against *S. aureus* [46]. It can be suspected that limonene may contribute to the antimicrobial action, together with the non-volatile components of the *J. communis* CO_2_ extract. 

*C. sativum* essential oil was found to be most active against *K. rhizophila* and *K. aerogenes* (MIC = 25 µg/mL). To our knowledge, the literature lacks studies on the activity of the main constituents of this raw material, namely 2(*E*)-decenal **9**, linalool **10**, 2-dodecen-1-ol **11**, and 2(*E*)-dodecenal **12**, against these microorganisms [35,36].

*A. balsamea* balsam absolute showed the highest inhibitory potency against *K. rhizophila, P. putida* (MIC = 12.5 µg/mL), *C. acnes*, and *K. aerogenes* (MIC = 25 µg/mL). This raw material mainly contains compounds such as β-pinene **13**, borneol acetate **14**, carene **15**, 8-hydroxylinalool **16**, camphene **17**, and α-pinene **18** in the volatile fraction [37]. To our knowledge, there are no studies demonstrating the antimicrobial activity of the individual chemical compounds mentioned against *K. rhizophila, P. putida, C. acnes,* and *K. aerogene*s. 

*C. officinalis* balsam showed the most prominent inhibitory effect against *K. rhizophila* (MIC = 25 µg/mL). The main volatile constituents of the raw material are β-caryophyllene (for which its antibacterial activity has already been described above) and α-copaene **19** [35,39]. However, consistent with our results are the results of Martins et al. who found that the essential oil of the inner bark of *Kielmetera coriacea*, of which α-copaene (14.9%) is one of the main components, showed positive antimicrobial activity against *Prevotella nigrescens* (ATCC 33563) (MIC = 25 µg/mL), but not against the microorganisms we studied [47].

Our results showed that *A. archangelica* essential oil is the most effective against *C. acnes* with an MIC = 25 µg/mL. This raw material mainly contains compounds such as α-pinene, β-phellandrene **20**, 3-carene, and limonene **21** in its composition [31]. Juliano et al. showed that *Santolina insularis* essential oil containing β-phellandrene (18.87%) inhibited the growth of *C. acnes* (ATCC 6919) with an MIC value of 1 mg/mL [48]. This is a higher result compared to that obtained in this study, but allows us to conclude that *A. archangelica* essential oil has antibacterial properties. The differences in the values presented may be due to the presence of other chemical compounds in this essential oil. 

Lastly, *P. cubeba* essential oil showed the highest inhibitory effect against *C. acnes* (MIC = 25 µg/mL) and *P. putida* (MIC = 12.5 µg/mL). The main constituents of this raw material are γ-cadinene **22**, β-cubebene **23**, and α-copaene [39]. The antibacterial effect of the raw materials, mainly γ-cadinene, in their composition has been confirmed against the species of *C. acnes* [49]. Furthermore, the growth inhibitory activity of the *P. putida* species was assessed using *Piper porphyrophyllum* essential oil containing α-copaene (13.2%) in its composition [50]. These results suggest that the described compounds significantly affect antimicrobial activity. The possible inconsistencies observed between our results and those reported by other investigators could be explained by differences in the experimental setup.

From the MIC values obtained in this study, all antibiotics showed antibacterial activity against different bacterial strains, but at different levels (the antibiotic results are presented in Table 3).

All bacteria tested were more or less sensitive to the three antibiotics. Differences may be due to evolved resistance mechanisms, the cell structure of the species, or the chemical structure of the antibiotic [50]. The MIC values obtained for antibiotics are lower than those for raw materials of natural fragrance. One way to achieve better results for essential oils is to use them in combination (synergism) [23,51,52]. This approach can reduce the required concentration of essential oils and improve their antimicrobial effectiveness. The combination of selected components that exhibit synergism will then reduce the concentration needed to achieve the same inhibitory effect against bacteria, compared to the use of individual components. The selection of suitable components depends on the MIC values they exhibit, their chemical structure, the percentage of their chemical composition, and, when given a fragrance raw material for combination, the effect on the bacterial cell and the main chemical compounds in the formulation. An example mechanism is to increase the permeability of the cytoplasmic membrane by one component while allowing the other component to be transported into the bacterial cell. To obtain satisfactory synergistic results, lower concentrations of components should be chosen whenever possible compared to their individual inhibitory concentrations. To test the effect of synergism, the checkerboard method can be used by placing medium, appropriately diluted fragrance raw materials and inoculum in a 96-well plate. As microorganisms are constantly developing resistance to conventional antibiotics, it is essential to explore alternative ways to combat them. Pathogenic bacterial species employed in this study have already developed resistance mechanisms against specific types of antibiotics [53,54,55,56,57,58,59,60,61,62]. However, most raw materials were able to inhibit their growth, indicating that the mechanisms responsible for antibiotic resistance may not guarantee resistance to natural fragrances. Although the tested raw materials may have a weaker effect on microorganism activity and growth compared to antibiotics, they exhibit great potential in the fight against pathogenic microorganisms and the treatment of bacterial infections caused by them. The results of the research obtained can contribute to the design of appropriate tools to combat resistant pathogens in the future in the pharmaceutical, cosmetic, or household industries.

## 4. Materials and Methods

### 4.1. Materials

The applied pathogenic bacteria strains, including *Staphylococcus aureus* WDCM 000322, WDCM 00193 (ATCC 6538), *Staphylococcus epidermidis* WDCM 00036 (ATCC 12228), *Kocuria rhizophila* ATCC 9341, *Klebsiella aerogenes* WDCM 00175 (ATCC 13048), *Klebsiella pneumoniae* WDCM 00097 (ATCC 13883), *Pluralibacter gergoviae* ATCC 33028, *Burkholderia cepacia* ATCC 25416, *Pseudomonas putida* ATCC 49128, *Pseudomonas fluorescens* WDCM 00115 (ATCC 13525), and *Cutibacterium acnes* ATCC 11827, were purchased from Sterbios, Poland.

Ninety-nine samples of natural fragrance materials were generously donated by the essential oil industry, members of the International Federation of Essential Oils and Aroma Trades, to ensure the highest quality. These materials included:

Lluch Essence (Spain): Essential oils of *Cinnamomum camphora*, *Lippia citriodora*, *Cananga odorata*, *Dipterocarpus* balsam, and *Copaifera officinalis* balsam, and oleoresins of *Ocimum basilicum*, *Capsicum annuum* var. *annuum* 1.000.000 SHU, *Capsicum annuum* 40.000 SHU, *Capsicum annuum* 80.000 SHU, *Piper nigrum* 40/20, *Thymus vulgaris*, *Origanum vulgare*, and *Laurus nobilis*.

Dutjahn Sandalwoods Oils (Australia): *Santalum spicatum* essential oil.

Vessel Essential Oils (Greece): Essential oil of *Ocimum basilicum* ct linalool.

A. Fakhry and Co. (Egypt): Essential oils of *Citrus* x *aurantium* var. *amara*, *Ocimum basilicum* ct methylchavicol, and *Tagetes minuta*, and absolute of *Cynara cardunculus*.

Albert Vieille (France): Absolute of *Cistus ladanifer*.

Eucaforest (Southern Africa): Essential oils of *Melaleuca alternifolia*, *Pelargonium graveolens*, *Eucalyptus smithii*, and *Leptospermum petersonii*.

Ultra International (India): Essential oils of *Angelica archangelica*, *Artemisia absinthium*, *Artemisia taurica*, *Citrus sinensis*, *Callitris intratropica*, *Fortunella japonica*, *Kunzea ambigua*, *Agathosma betulina*, *Eremophila mitchellii*, *Chamaecyparis obtusa*, *Coriandrum sativum*, *Backhousia citriodora*, *Eucalyptus kochii*, and *Melaleuca ericifolia*, and CO_2_ extracts of *Juniperus communis*, *Coffea canephora*, *Coffea arabica*, *Chrysopogon zizanioides*, *Elettaria cardamomum*, *Nigella sativa*, *Curcuma longa*, *Illicium verum*, and *Zingiber officinale*.

Berje Inc. (USA): Essential oils of *Angelica archangelica* root, *Angelica archangelica* seed, *Illicium verum*, *Aniba rosodora*, *Agathosma betulina*, *Agathosma crenulate*, *Acorus calamus*, *Croton eluteria* bark, *Nepeta cataria*, *Coffea arabica*, *Vitis vinifera*, *Piper cubeba*, *Anethum graveolens* seed, *Abies balsamea* needle, *Lavandula angustifolia*, *Levisticum officinale* leaf, *Levisticum officinale* root, *Citrus reticulata*, *Achillea millefolium*, *Ocotea cymbarum*, *Petroselinum crispum* leaf, *Mentha* x *piperita* Yakima, *Perilla frutescens*, *Pinus pumilio*, *Rosa damascene* Bulgaria and Turkey, *Sassafras albidum*, *Satureja hortensis*, *Fokienia hodginsii*, *Tagetes minuta*, *Valeriana officinalis* root, and *Artemisia absinthium* European, and absolutes of *Abies balsamea*, *Rosa damascena* Morocco and Bulgaria, *Picea glauca*, and *Abies balsamea* concrete, *Iris pallida* concentrate, and refined gum of *Cistus ladanifer* and *Liquidambar styraciflua*.

Lebermuth (USA): Essential oils of *Cedrus deodora* Himalayan, *Eucalyptus polybractea*, *Solidago canadensis*, *Citrus x paradisi*, *Mentha* x *piperita* tails, *Picea mariana*, *Melaleuca alternifolia*, *Cananga odorata*, *Myroxylon peruiferum* resin, and *Azadirachta indica* oil.

Microplates were obtained from NEST Biotechnology Co., Ltd., China. Nutrient LAB-AGAR^TM^, Brain Heart Infusion LAB-AGAR^TM^, Brain Heart Infusion BrothTM and Nutrient Broth^TM^ were provided by BioMaxima S.A. (Poland). The alamarBlue^TM^ reagent was acquired from Bio-Rad Antibodies UK. Dimethyl sulfoxide was purchased from Honeywell Sp. z.o.o. The alamarBlue^TM^ reagent was obtained from Bio-Rad-Antibodies UK. The antimicrobial agents gentamicin, ciprofloxacin, and ampicillin were purchased from Merck Poland.

### 4.2. Methods

#### 4.2.1. Test Microorganisms

All bacterial strains were subcultured from the original culture (KWIK-STIK) and kept in Nutrient LAB-AGAR^TM^ or Brain Heart Infusion Broth AGAR^TM^ plates at 4 °C and grown in the appropriate conditions and medium presented in Table 4.

#### 4.2.2. Natural Fragrance Materials

Natural fragrance raw materials with an initial concentration of 10 mg/mL in DMSO were used. DMSO at low concentrations does not have a significant effect on the growth in short-term experiments [63]. In this study, the 8% DMSO concentration used was not toxic to bacterial strains.

#### 4.2.3. Antibacterial Activity Screening Assay

The purpose of the screening tests was to identify compounds that inhibit the growth of microorganisms. Furthermore, screening tests allow to determine cytotoxicity, early safety screening, and antimicrobial potential of minor oxime constituents of natural raw materials. All tested bacterial strains were subcultured in appropriate medium and incubated under optimal conditions. Cell cultures were used to make suspensions in physiological saline corresponding to the McFarland protocol to obtain suspensions of approximately 1–2 × 10^8^ CFU/mL [64]. The suspensions obtained were used to inoculate medium to achieve the final cell concentration of 5 × 10^6^ CFU/mL (OD_550_ = 0.125).

The appropriate medium was added to the 96-well plates in volumes: col. 1–9: 82 µL, col. 10: 90 µL, col. 11: 100 µL, and col. 12: 110 µL. Natural fragrance materials dissolved in DMSO were placed in 96-well plates in col. 1–9 in triplicate (final concentration 400 µg/mL). The inoculum was added to the col. 1–11 in a volume of 10 µL, and plates were incubated under the appropriate conditions for each of the strains according to Table 4. After incubation, 10 µL of alamarBlue^TM^ reagent was added to the col. 1–11. The plates were again incubated for 3 h under appropriate conditions and then analysed (the procedure is described in Section 4.2.5). The 96-well plates included one control with DMSO in col. 10 (medium + inoculum + 8% DMSO solution), one positive control in col. 11 (medium + inoculum + antibiotic), and one negative control in col. 12 (medium + inoculum). In the tests, antibiotics used gentamicin at concentration of 20 µg/mL for *S. aureus*, *K. rhizophila*, *P. fluorescens*, and *P. putida*; ciprofloxacin at concentration of 10 µg/mL for *K. rhizophila*, *P. fluorescens*, *P. putida*, *B. cepacia*, *K. aerogenes*, and *K. pneumoniae*; and ampicillin at concentration 2 µg/mL for *S. epidermidis*, *C. acnes*, and *P. gergoviae*.

#### 4.2.4. Minimal Inhibitory Concentration (MIC)

The objective of the tests was to determine the minimum inhibitory concentration (MIC) of the natural fragrance materials against the microorganisms tested. Cell density was obtained in a way similar to that of the screening test. Natural fragrance materials dissolved in DMSO were diluted (two times) in 96-well plates in the concentration range of 800–3.125 µg/mL for all bacteria. The appropriate medium was added to the 96-well plates in volumes: col. 1: 164 µL, col. 2–10: 90 µL, col. 11: 100 µL, and col. 12: 110 µL. The natural material was added to the col. 1 in volume of 16 µL, and two-fold serial dilutions were prepared horizontally on the plate. Excess dilutions (90 µL) were discarded from the plate. The subsequent part of the MIC assay was carried out in the same way as for the screening tests (the procedure is described in Section 4.2.3).

#### 4.2.5. Analysis of the Screening and MIC Assays

The basis for the change in the analysis of the assays was the colour of the alamarBlue^TM^ reagent from pink to blue. The lack of colour change after the addition of the dye indicated inhibition of bacterial growth, as described by Rampersad [65]. The MIC value was determined as the last well showing a noticeable blue colour, expressed in µg/mL. Screening and MIC assays were performed and analysed according to CLSI (Clinical and Laboratory Standards Institute) with slight modifications [66,67].

## 5. Conclusions

The growth inhibitory and microbial activity of the 99 fragrance materials was determined for the first time against specific bacterial species, namely *S. aureus* (ATCC 6538), *S. epidermidis* (ATCC 12228), *K. rhizophila* (ATCC 9341), *K. aerogenes* (ATCC 13048), *K. pneumoniae* (ATCC 13883), *P. gergoviae* (ATCC 33028), *B. cepacia* (ATCC 25416), *P. putida* (ATCC 49128), *P. fluorescens* (ATCC 13525), and *C. acnes* (ATCC 11827). Screening tests revealed that *S. epidermidis* was the most resistant Gram-positive bacterium, inhibited by 22 fragrance materials, whereas *P. gergoviae* was the most resistant Gram-negative bacterium, inhibited by 11 materials. The appropriate tests demonstrated that the lowest MIC values were obtained for *S. aureus* (*Nigella sativa* expressed oil, *Cynara scolymus* absolute, MIC = 25 µg/mL), *K. rhizophila* (*Abies balsamea* absolute, MIC = 12.5 µg/mL), and *P. putida* (*Piper cubeba* EO, *Abies balsamea* absolute, MIC = 12.5 µg/mL). On the basis of the results obtained, it indicates the antibacterial potential of natural fragrance raw materials. We can conclude that the most active aroma materials against the pathogens tested offer an alternative to chemical preservatives and antibiotics. Thus, they can be used as ingredients in various formulations of the cosmetic, chemical, medical, or food industries. In addition to providing unique characteristics such as fragrance and colour, they will exhibit antimicrobial activity to prevent product contamination.

## Figures and Tables

**Table 1:** Minimal inhibitory concentration (MIC) values for the most active fragrance raw materials against all strains evaluated.

No.	English Common Name	Botanical Name	Country of Origin	MIC [µg/mL]
***Staphylococcus aureus***				
**1**	Black seed expressed EO	*Nigella sativa* L.	India	25
**2**	Blue cypress EO	*Callitris intratropica* Baker and H.G.Sm.	Australia	50
**3**	Pepper black oleoresin 40/20	*Piper nigrum* L.	India	100
**4**	Copaiba balsam	*Copaifera officinalis* L.	Brazil	100
**5**	Hinoki EO	*Chamaecyparis obtusa* (Siebold and Zucc.) Endl.	Japan	100
**6**	Cognac white EO	*Vitis vinifera* L.	USA	73.17
**7**	Orris root concentrate	*Iris pallida* Lam.	France	73.17
***Staphylococcus epidermidis***				
**8**	Juniper berry CO_2_	*Juniperus communis* L.	India	25
**9**	Coriander herb EO	*Coriandrum sativum* L.	Russia	50
**10**	Blue cypress EO	*Callitris intratropica* Baker and H.G.Sm.	Australia	50
**11**	Fir balsam Canadian absolute	*Abies balsamea* (L.) Mill.	Canada	50
**12**	Sandalwood EO	*Santalum spicatum* (R.Br.) A. DC	Australia	100
**13**	Vetiver CO_2_	*Chrysopogon zizanioides* (L.) Roberty syn. *Vetiveria zizanioides* (L.) Nash	India	100
**14**	Ginger CO_2_	*Zingiber officinale* Roscoe	India	100
**15**	Fir balsam Canadian concrete	*Abies balsamea* (L.) Mill.	Canada	100
**16**	Labdanum gum refined	*Cistus ladanifer* L.	Spain	100
***Kocuria rhizophila***				
**17**	Fir balsam Canadian absolute	*Abies balsamea* (L.) Mill.	Canada	12.5
**18**	Copaiba balsam	*Copaifera officinalis* L.	Brazil	25
**19**	Juniper berry CO_2_	*Juniperus communis* L.	India	25
**20**	Coriander herb EO	*Coriandrum sativum* L.	Russia	25
**21**	Blue cypress EO	*Callitris intratropica* Baker and H.G.Sm.	Australia	50
**22**	Mugwort EO	*Artemisia taurica* Willd.	Russia	100
***Cutibacterium acnes***				
**23**	Angelica root EO	*Angelica archangelica* L.	Hungary	25
**24**	Cubeb EO	*Piper cubeba* L.f.	Singapore	25
**25**	Fir balsam Canadian absolute	*Abies balsamea* (L.) Mill.	Canada	25
**26**	Catnip (catmint) EO	*Nepeta cataria* L.	Canada	100
**27**	Fir balsam Canadian concrete	*Abies balsamea* (L.) Mill.	Canada	100
***Pluralibacter gergoviae***				
**28**	Juniper berry CO_2_	*Juniperus communis* L.	India	50
**29**	Fir balsam Canadian absolute	*Abies balsamea* (L.) Mill.	Canada	100
***Pseudomonas fluorescens***				
**30**	Juniper berry CO_2_	*Juniperus communis* L.	India	50
**31**	Fir balsam Canadian absolute	*Abies balsamea* (L.) Mill.	Canada	50
**32**	Ginger CO_2_	*Zingiber officinale* Roscoe	India	100
**33**	Blue cypress EO	*Callitris intratropica* Baker and H.G.Sm.	Australia	100
**34**	Coffee EO	*Coffea arabica* L.	Canada	100
**35**	Cognac white EO	*Vitis vinifera* L.	USA	100
***Pseudomonas putida***				
**36**	Cubeb EO	*Piper cubeba* L.f.	Singapore	12.5
**37**	Fir balsam Canadian absolute	*Abies balsamea* (L.) Mill.	Canada	12.5
**38**	Blue cypress EO	*Callitris intratropica* Baker and H.G.Sm.	Australia	25
**39**	Fir balsam Canadian concrete	*Abies balsamea* (L.) Mill.	Canada	50
**40**	Juniper Berry CO_2_	*Juniperus communis* L.	India	100
**41**	Vetiver CO_2_	*Chrysopogon zizanioides* (L.) Roberty syn. *Vetiveria zizanioides* (L.) Nash	India	100
**42**	Hinoki EO	*Chamaecyparis obtusa* (Siebold and Zucc.) Endl.	Japan	100
***Burkholderia cepacia***				
**43**	Blue cypress EO	*Callitris intratropica* Baker and H.G.Sm.	Australia	25
**44**	Juniper berry CO_2_	*Juniperus communis* L.	India	50
**45**	Coriander herb EO	*Coriandrum sativum* L.	Russia	50
**46**	Fir balsam Canadian absolute	*Abies balsamea* (L.) Mill.	Canada	50
**47**	Vetiver CO_2_	*Chrysopogon zizanioides* (L.) Roberty syn. *Vetiveria zizanioides* (L.) Nash	India	100
**48**	Fir balsam Canadian concrete	*Abies balsamea* (L.) Mill.	Canada	100
***Klebsiella aerogenes***				
**49**	Juniper berry CO_2_	*Juniperus communis* L.	India	25
**50**	Coriander herb EO	*Coriandrum sativum* L.	Russia	25
**51**	Fir balsam Canadian absolute	*Abies balsamea* (L.) Mill.	Canada	25
**52**	Blue cypress EO	*Callitris intratropica* Baker and H.G.Sm.	Australia	50
**53**	Labdanum gum refined	*Cistus ladanifer* L.	Spain	100
**54**	Artichoke absolute	*Cynara scolymus* L.	Egypt	100
***Klebsiella pneumoniae***				
**55**	Coriander herb EO	*Coriandrum sativum* L.	Russia	50
**56**	Juniper berry CO_2_	*Juniperus communis* L.	India	100
**57**	Blue cypress EO	*Callitris intratropica* Baker and H.G.Sm.	Australia	100
**58**	Fir balsam Canadian absolute	*Abies balsamea* (L.) Mill.	Canada	100

**Table 2:** Chemical composition of selected natural fragrance raw materials showing the lowest MIC values.

Botanical Name of the Plant	Natural Raw Material	Plant Parts	Major Components	Geographical Sources (Region)	The Lowest MIC Values	Ref.	Producer
*Nigella sativa* L.	Black seed expressed oil	seeds	cuminaldehyde **1** (20–50%) β- caryophyllene **2** (0.1–1%)	India	*S. aureus* (MIC = 25 µg/mL)	[30]	Ultra International B.V.
*Callitris intratropica* Baker and H.G.Sm.	Blue cypress essential oil	chipped bark, wood	guaiol **3** (14.64%), bulnesol **4** (10.58%), dihydrocolumellarin **5** (10.12%), γ-eudesmol **6** (8.40%)	Australia	*P. putida, * *B. cepacia* (MIC = 25 µg/mL)	[31,32]	Ultra International B.V.
*Juniperus communis* L.	Juniper berry essential oil	ripe berries	(-)- α-pinene **7** (44.47%), D-limonene **8** (19.41%)	India	*K. rhizophila, S.epidermidis, K. aerogenes* (MIC = 25 µg/mL)	[33,34]	Ultra International B.V.
*Coriandrum sativum* L.	Coriander herb essential oil	leaves	2(*E*)-decenal **9** (27.78%), (-)-linalool **10** (18.72%), 2-dodecen-1-ol **11** (18.53%), 2(*E*)-dodecenal **12** (6.19%)	Russia	*K. rhizophila, K. aerogenes* (MIC = 25 µg/mL)	[35,36]	Ultra International B.V.
*Abies balsamea* (L.) Mill.	Fir balsam absolute	needle-like leaves	β-pinene **13** (22.5 ± 0.44%), borneol acetate **14** (18.0 ± 0.23%), 3-carene **15** (10.3 ± 0.15%), 8-hydroxylinalool **16** (9.34 ± 0.11%), camphene **17** (7.98 ± 0.15%), α-pinene **18** (7.09 ± 0.18%)	Canada	*K. rhizophila, P. putida* (MIC = 12.5 µg/mL) *C. acnes, * *K. aerogenes *(MIC = 25 µg/mL)	[37]	Berje Inc.
*Copaifera officinalis* L.	Copaiba balsam	tree trunk resin	β-caryophyllene **2** (35.03%) α-copaene **19** (33.61%)	Brazil	*K. rhizophila (*MIC = 25 µg/mL)	[38]	Lluch Essence
*Angelica archangelica* L.	Angelica root essential oil	root	α-pinene **18** (20–25%), 3-carene **15** (12–17%), β-phellandrene **20** (6.0–15%), limonene **21** (5.0–15%)	France	*C. acnes* (MIC = 25 µg/mL)	[31]	Ultra International B.V.
*Piper cubeba* L.f.	Cubeb essential oil	unripeberries	γ –cadinene **22** (13 ± 0.17%), β-cubebene **23** (12.1 ± 0.62%), α-copaene **19** (11.7 ± 0.17%)	Singapore	*P. putida* (MIC = 12.5 µg/mL) *C. acnes* (MIC = 25 µg/mL)	[39]	Berje Inc.

**Table 3:** Results for antibiotics obtained for tested bacterial strains.

Bacteria		Antibiotics [μg/mL]		
		Gentamicin	Ciprofloxacin	Ampicillin
Gram-positive	*S. aureus*	5.000		
	*K. rhizophila*	1.000	0.625	
	*S. epidermidis*			0.0625
	*C. acnes*			0.250
Gram-negative	*P. gergoviae*			0.008
	*P. fluorescens*	2.000	0.008	
	*P. putida*	0.625	0.019	
	*B. cepacia*		0.250	
	*K. aerogenes*		0.019	
	*K. pneumoniae*		0.500	

**Table 4:** Incubation conditions for selected bacteria (conditions provided by ATCC).

Bacteria Name + Trade Name	Incubation Temperature [^o^ C]	Incubation Time [h]	Medium	Environment
*Staphylococcus aureus* WDCM 000322, WDCM 00193 (ATCC 6538)	37	48	Nutrient Broth	Aerobic
*Staphylococcus epidermidis* WDCM 00036 (ATCC 12228)				
*Kocuria rhizophila* (ATCC 9341)	30	24		
*Klebsiella aerogenes* WDCM 00175 (ATCC 13048)				
*Klebsiella pneumoniae* WDCM 00097 (ATCC 13883)	37			
*Pluralibacter gergoviae* (ATCC 33028)				
*Burkholderia cepacia* (ATCC 25416)	30	48		
*Pseudomonas putida* (ATCC 49128)				
*Pseudomonas fluorescens* WDCM 00115 (ATCC 13525)	26			
*Cutibacterium * *Acne*s (ATCC 11827)	37	24	Brain Heart Infusion Broth	Anaerobic

## Data Availability

Not applicable.

## Supplementary Materials

The following supporting information can be downloaded at: https://www.mdpi.com/article/10.3390/plants12213777/s1, Table S1. Minimal Inhibitory Concentration (MIC) parameters of natural flavour and fragrance materials on all evaluated strains.
