# Supplementary material for: Exploring the Antimicrobial Properties of 99 Natural Flavour and Fragrance Raw Materials against Pathogenic Bacteria: A Comparative Study with Antibiotics
Source: Plants (Basel). 2023 Nov 6;12(21):3777. doi: 10.3390/plants12213777 (PMC10648197; doi:10.3390/plants12213777)
Supplement: Supplementary file 1 [file plants-12-03777-s001.zip › plants-2671291-supplementary.pdf]

# Exploring the Antimicrobial Properties of 99 Natural Flavor and Fragrance Raw Materials Against Pathogenic Bacteria: A Comparative Study with Antibiotics

Bacińska Zuzanna[1,2], Baberowska Kinga[1,3], Surowiak Alicja Karolina[1], Balcerzak Lucyna[1], Strub Daniel Jan[1,*]

[1]Department of Chemical Biology and Bioimaging, Faculty of Chemistry, Wrocław University of Science and Technology, Wyb. Wyspiańskiego 27, 50-370 Wrocław, Poland

[2]Department of Engineering and Technology of Chemical Processes, Faculty of Chemistry, Wrocław University of Science and Technology, Wyb. Wyspiańskiego 27, 50-370 Wrocław, Poland

[3]Department of Analytical Chemistry and Chemical Metallurgy, Faculty of Chemistry, Wrocław University of Science and Technology, Wyb. Wyspiańskiego 27, 50-370 Wrocław, Poland

e-mail: daniel.strub@pwr.edu.pl

Table S1. Minimal Inhibitory Concentration (MIC) parameters of natural flavour and fragrance materials on all evaluated strains.

| No. | English common name of natural fragrance material | Company | CAS number | Botanical name | Origin | Gram-positive bacteria | | | | Gram-negative bacteria | | | | | |
|---|---|---|---|---|---|---|---|---|---|---|---|---|---|---|---|
| | | | | | | *S. aureus* | *K. rhizophila* | *S. epidermidis* | *C. acnes* | *P. gergoviae* | *P. fluorescens* | *P. putida* | *B. cepacia* | *K. aerogenes* | *K. pneumoniae* |
| | | | | | | MIC assay [µg/ml] | MIC assay [µg/ml] | MIC assay [µg/ml] | MIC assay [µg/ml] | MIC assay [µg/ml] | MIC assay [µg/ml] | MIC assay [µg/ml] | MIC assay [µg/ml] | MIC assay [µg/ml] | MIC assay [µg/ml] |
| *Achariaceae* | | | | | | | | | | | | | | | |
| 1. | Styrax gum | Berje Inc. | 8046-19-3 | *Liquidambar styraciflua* L. | Honduras | >400 | >400 | 800 | >400 | >400 | >400 | >400 | >400 | >400 | >400 |
| *Acoraceae* | | | | | | | | | | | | | | | |
| 2. | Calamus EO | Berje Inc. | 8015-79-0 | *Acorus calamus* L. | Nepal | 400 | 800 | >400 | 800 | >400 | 200 | >400 | >400 | 800 | >400 |
| *Annonaceae* | | | | | | | | | | | | | | | |
| 3. | Ylang-Ylang I EO | Lluch Essence | 8006-81-3 | *Cananga odorata* Hook. f. & Thomson forma *genuina* | Comoros | >400 | >400 | >400 | >400 | >400 | >400 | >400 | >400 | >400 | >400 |
| 4. | Ylang-Ylang I EO | Lebermuth | 8006-81-3 | | Madagascar | 800 | >400 | >400 | >400 | >400 | >400 | >400 | >400 | >400 | >400 |
| 5. | Ylang-Ylang II EO | Lebermuth | 8006-81-3 | | Madagascar | 800 | >400 | >400 | >400 | >400 | >400 | >400 | >400 | >400 | >400 |
| 6. | Ylang-Ylang III EO | Lluch Essence | 8006-81-3 | | Comoros | >400 | >400 | >400 | >400 | >400 | >400 | >400 | >400 | >400 | >400 |
| *Apiaceae* | | | | | | | | | | | | | | | |
| 7. | Angelica root Oil | Ultra International | 8015-64-3 | *Angelica archangelica* L. | France | 400 | >400 | >400 | 800 | >400 | >400 | >400 | >400 | 800 | >400 |
| 8. | Angelica root EO | Berje Inc. | 8015-64-3 | | Hungary | >400 | 400 | >400 | 25 | >400 | 200 | 200 | 800 | 800 | >400 |
| 9. | Angelica seed EO | Berje Inc. | 8015-64-3 | | USA | >400 | 400 | >400 | 800 | >400 | 200 | 200 | 400 | >400 | >400 |
| *Apiaceae* | | | | | | | | | | | | | | | |
| 10. | Coriander herb EO | Ultra International | 8008-52-4 | *Coriandrum sativum* L. | Russia | >400 | 25 | 50 | 800 | 200 | 200 | 200 | 50 | 25 | 50 |
| 11. | Dill seed EO | Berje Inc. | 8006-75-5 | *Anethum graveolens* L. | South Africa | 200 | >400 | >400 | >400 | >400 | >400 | >400 | >400 | >400 | >400 |
| 12. | Lovage leaf EO | Berje Inc. | 8016-31-7 | *Levisticum officinale* W.D.J. Koch | Hungary | >400 | >400 | >400 | >400 | >400 | >400 | >400 | >400 | >400 | >400 |
| 13. | Lovage root EO | Berje Inc. | 8016-31-7 | | Hungary | 200 | 800 | >400 | >400 | >400 | 400 | 800 | >400 | >400 | >400 |
| 14. | Parsley leaf EO | Berje Inc. | 8000-68-8 | *Petroselinum crispum* (Mill.) Fuss syn. *Petroselinum sativum* Hoffm | Australia | 400 | 800 | >400 | >400 | >400 | 200 | 400 | 800 | >400 | >400 |
| *Asteraceae* | | | | | | | | | | | | | | | |
| 15. | Artichoke absolute | A. Fakhry & Co. | 93165-00-5 | *Cynara cardunculus* L. | Egypt | >400 | >400 | >400 | 800 | >400 | >400 | 400 | 800 | 100 | >400 |
| 16. | Goldenrod EO | Lebermuth | 96690-40-3 | *Solidago canadensis* L. | Canada | >400 | >400 | >400 | >400 | >400 | >400 | 400 | >400 | >400 | >400 |
| 17. | Milfoil EO | Berje Inc. | 8022-07-9 | *Achillea millefolium* L. | Spain | >400 | >400 | >400 | >400 | >400 | >400 | >400 | >400 | >400 | >400 |
| 18. | Mugwort EO | Ultra International | 8008-93-3 | *Artemisia taurica* Willd. | Russia | >400 | 100 | >400 | >400 | >400 | >400 | >400 | >400 | >400 | >400 |
| 19. | Tagete EO | A. Fakhry & Co. | 8016-84-0 | *Tagetes minuta* L. syn. *Tagetes glandulifera* Schrank | Egypt | >400 | >400 | >400 | >400 | >400 | 200 | >400 | >400 | >400 | >400 |
| 20. | Tagete EO | Berje Inc. | 8016-84-0 | | Zimbabwe | 400 | >400 | 800 | >400 | >400 | 200 | 400 | >400 | >400 | >400 |
| 21. | Wormwood herb EO | Berje Inc. | 8008-93-3 | *Artemisia absinthium* L. | Russia | >400 | >400 | >400 | >400 | >400 | >400 | >400 | >400 | >400 | >400 |
| 22. | Wormwood herb EO | Berje Inc. | 8008-93-3 | *Artemisia absinthium* L. | USA | >400 | >400 | >400 | >400 | >400 | >400 | >400 | >400 | >400 | >400 |
| *Cistaceae* | | | | | | | | | | | | | | | |
| 23. | Cistus SEV absolute | Albert Vieille | 8016-26-0 | *Cistus ladanifer* L. | Spain | >400 | >400 | >400 | >400 | >400 | >400 | >400 | >400 | >400 | >400 |
| 24. | Labdanum gum refined | Berje Inc. | 8016-26-0 | | Spain | >400 | 200 | 100 | 800 | 800 | 200 | 200 | 200 | 100 | 400 |
| *Cupressaceae* | | | | | | | | | | | | | | | |
| 25. | Blue cypress EO | Ultra International | 187348-13-6 | *Callitris intratropica* Baker & H.G.Sm. | Australia | 50 | 50 | 50 | 400 | 200 | 100 | 25 | 25 | 50 | 100 |
| 26. | Hinoki EO | Ultra International | 91745-97-0 | *Chamaecyparis obtusa* (Siebold & Zucc.) Endl. | Japan | 100 | 200 | 400 | 800 | >400 | 200 | 100 | 200 | 400 | >400 |
| 27. | Juniper berry $CO_2$ | Ultra International | 8012-91-7 | *Juniperus communis* L. | India | 200 | 25 | 25 | 800 | 50 | 50 | 100 | 50 | 25 | 100 |
| 28. | Siam wood EO | Berje Inc. | 8022-47-7 | *Fokienia hodginsii* (Dunn) A.Henry & H H.Thomas | Vietnam | >800 | 800 | 800 | 800 | >400 | >400 | 400 | 800 | 800 | 800 |
| *Dipterocarpaceae* | | | | | | | | | | | | | | | |
| 29. | Gurjun Balsam rectified (Copaene) | Lluch Essence | 8030-55-5 | *Dipterocarpus* C. F. Gaertner | Indonesia | >400 | >400 | >400 | >400 | >400 | >400 | 400 | >400 | >400 | >400 |
| *Euphorbiaceae* | | | | | | | | | | | | | | | |
| 30. | Cascarilla bark EO | Berje Inc. | 28231-03-0 | *Croton eluteria* (L.) W. Wright syn. *Clutia eluterzia* L. | El Salvador | >400 | 200 | 200 | 800 | >400 | >400 | 200 | 400 | 400 | >400 |
| *Fabaceae* | | | | | | | | | | | | | | | |
| 31. | Copaiba Balsam | Lluch Essence | 8001-61-4 | *Copaifera officinalis* L. | Brazil | 100 | 25 | 200 | >400 | 800 | >400 | 400 | 200 | 800 | >400 |
| 32. | Peru balsam resin | Lebermuth | 8007-00-9 | *Myroxylon peruiferum* L.f. | Peru | >400 | >400 | >400 | >400 | >400 | >400 | >400 | >400 | >400 | >400 |
| *Geraniaceae* | | | | | | | | | | | | | | | |
| 33. | Rose geranium EO | Eucaforest | 90082-55-6 | *Pelargonium graveolens* L'Hér. | South Africa | >400 | >400 | >400 | >400 | >400 | 400 | 800 | >400 | >400 | >400 |
| *Iridaceae* | | | | | | | | | | | | | | | |
| 34. | Orris root concentrate | Berje Inc. | 8002-73-1 | *Iris pallida* Lam. | France | 100 | 400 | 400 | 800 | 800 | >400 | >400 | 800 | 400 | 800 |
| *Lamiaceae* | | | | | | | | | | | | | | | |

| | | | | | | | | | | | | | | | |
|---|---|---|---|---|---|---|---|---|---|---|---|---|---|---|---|
| 35. | Basil oleoresin | Lluch Essence | 8015-73-4 | *Ocimum basilicum* L. | India | 800 | 800 | >400 | >400 | >400 | >400 | 800 | 800 | 800 | >400 |
| 36. | Basil sweet CT (linalool) EO | Vessel Essential Oils | 8015-73-4 | | Greece | >400 | >400 | >400 | >400 | >400 | >400 | >400 | >400 | >400 | >400 |
| 37. | Basil tropical CT (methylchavicol) EO | A. Fakhry & Co. | 8015-73-4 | | Egypt | >400 | >400 | >400 | >400 | >400 | >400 | >400 | >400 | >400 | >400 |
| 38. | Catnip (catmint) EO | Berje Inc. | 8023-84-5 | *Nepeta cataria* L. | Canada | >400 | 800 | >400 | 100 | >400 | 200 | 400 | >400 | >400 | >400 |
| 39. | Lavandin abrialis EO | Berje Inc. | 8022-15-9 | *Lavandula angustifolia* Mill. × *Lavandula latifolia* Medik. | France | >400 | >400 | >400 | >400 | >400 | >400 | >400 | >400 | >400 | >400 |
| 40. | Origanum oleoresin | Lluch Essence | 8007-11-2 | *Origanum vulgare* L. | Turkey | 400 | >400 | >400 | >400 | >400 | >400 | >400 | >400 | >400 | >400 |
| 41. | Peppermint Yakima EO | Berje Inc. | 8006-90-4 | *Mentha* x *piperita* L. | USA | >400 | >400 | >400 | >400 | >400 | >400 | >400 | >400 | >400 | >400 |
| 42. | Peppermint tails EO | Lebermuth | N/A | | Italy | >400 | >400 | >400 | >400 | >400 | >400 | >400 | >400 | >400 | >400 |
| 43. | Perilla EO | Berje Inc. | 68132-21-8 | *Perilla frutescens* (L.) Britton | China | >400 | >400 | >400 | >400 | >400 | >400 | >400 | >400 | >400 | >400 |
| 44. | Savory summer EO | Berje Inc. | 8016-68-0 | *Satureja hortensis* L. | Hungary | >400 | 800 | >400 | >400 | >400 | 400 | 800 | 800 | >400 | >400 |
| 45. | Thyme red oleoresin | Lluch Essence | 8007-46-3 | *Thymus vulgaris* L. | Turkey | 400 | >400 | >400 | >400 | >400 | >400 | 400 | >400 | >400 | >400 |
| *Lauraceae* | | | | | | | | | | | | | | | |
| 46. | Laurel Oleoresin | Lluch Essence | 8007-48-5 | *Laurus nobilis* L. | Spain | 400 | 800 | >400 | 800 | >400 | >400 | 800 | 800 | 800 | 800 |
| 47. | *Ocotea cymbarum* EO | Berje Inc. | 68917-09-9 | *Ocotea cymbarum* Kunth | China | >400 | >400 | >400 | >400 | >400 | >400 | >400 | >400 | >400 | >400 |
| 48. | Ravintsara EO | Lluch Essence | 92201-50-8 | *Cinnamomum camphora* Ness et Eberm. | Madagascar | >400 | >400 | >400 | >400 | >400 | >400 | >400 | >400 | >400 | >400 |
| 49. | Rosewood (Bois de rose) EO | Berje Inc. | 8015-77-8 | *Aniba rosodora* Ducke | India | >400 | >400 | >400 | >400 | >400 | 200 | >400 | >400 | >400 | >400 |
| 50. | Sassafras EO | Berje Inc. | 8006-80-2 | *Sassafras albidum* (Nutt.) Nees | Taiwan | >400 | >400 | >400 | >400 | >400 | >400 | >400 | >400 | >400 | >400 |
| *Meliaceae* | | | | | | | | | | | | | | | |
| 51. | Neem oil (exp.) | Lebermuth | 8002-65-1 | *Azadirachta indica* A. Juss. | India | 800 | >400 | >400 | >400 | >400 | >400 | >400 | >400 | >400 | >400 |
| *Myrtaceae* | | | | | | | | | | | | | | | |
| 52. | Blue mallee EO | Lebermuth | 91771-67-4 | *Eucalyptus polybractea* R. T. Baker | Australia | >400 | >400 | >400 | >400 | >400 | >400 | >400 | >400 | >400 | >400 |
| 53. | *Eucalyptus kochii* EO | Ultra International | 97926-42-6 | *Eucalyptus kochii* Maiden & Blakely | Australia | >400 | >400 | >400 | >400 | >400 | 200 | >400 | >400 | >400 | >400 |
| 54. | *Eucalyptus smithii* EO | Eucaforest | 91771-68-5 | *Eucalyptus smithii* R. T. Baker | South Africa | >400 | >400 | >400 | >400 | >400 | >400 | >400 | >400 | >400 | >400 |
| 55. | Kunzea EO | Ultra International | 97553-36-1 | *Kunzea ambigua* (Sm.) Druce | Australia | >400 | >400 | >400 | >400 | >400 | >400 | 400 | >400 | >400 | >400 |
| 56. | Lemon Myrtle EO | Ultra International | 84775-80-4 | *Backhousia citriodora* F. Muell. | Australia | 400 | >400 | >400 | >400 | >400 | 400 | 800 | >400 | >400 | >400 |
| 57. | Lemon scented tea tree EO | Eucaforest | 85085-43-4 | *Leptospermum petersonii* F. M. Bailey syn. *Leptospermum citratum* Challinor, Cheel & Penfold | South Africa | >400 | >400 | >400 | >400 | >400 | >400 | >400 | >400 | >400 | >400 |
| 58. | Rosalina EO | Ultra International | 68647-73-4 | *Melaleuca ericifolia* Sm. | Australia | >400 | 200 | >400 | 800 | >400 | 200 | 200 | 200 | >400 | >400 |
| 59. | Tea tree EO | Lebermuth | 68647-73-4 | *Melaleuca alternifolia* (Maiden & Betche) Cheel | China | >400 | >400 | >400 | >400 | >400 | >400 | >400 | >400 | >400 | >400 |
| 60. | Tea tree EO | Eucaforest | 68647-73-4 | | South Africa | >400 | >400 | >400 | >400 | >400 | 200 | 200 | >400 | >400 | >400 |
| *Pinaceae* | | | | | | | | | | | | | | | |
| 61. | Cedarwood Himalayan EO | Lebermuth | 68991-36-6 | *Cedrus deodora* (Roxb. ex Lamb.) G.Don | India | >400 | >400 | >400 | >400 | >400 | >400 | 400 | >400 | >400 | >400 |
| 62. | Fir needle EO | Berje Inc. | 8024-15-5 | *Abies balsamea* (L.) Mill. | Canada | 400 | >400 | >400 | >400 | >400 | 200 | 200 | >400 | >400 | >400 |
| 63. | Fir balsam absolute | Berje Inc. | 8024-15-5 | | Canada | 200 | 12.5 | 50 | 25 | 100 | 50 | 12.5 | 50 | 25 | 100 |
| 64. | Fir balsam concrete | Berje Inc. | 8024-15-5 | | Canada | 400 | 200 | 100 | 100 | >400 | >400 | 50 | 100 | 200 | 400 |
| 65. | Pine pumilio EO | Berje Inc. | 616-768-0 | *Pinus pumilio* Turra | Germany | >400 | >400 | >400 | >400 | >400 | 200 | 800 | >400 | >400 | >400 |
| 66. | Spruce absolute | Berje Inc. | 294-420-3 | *Picea glauca/Picea mariana* (Mill.) Britton, Sterns & Poggenb. | Canada | >400 | 800 | 800 | >400 | >400 | >400 | 400 | 800 | 800 | 800 |
| 67. | Spruce black EO | Lebermuth | 91722-19-9 | *Picea mariana* (Mill.) Britton, Sterns & Poggenb. | Canada | >400 | >400 | >400 | >400 | >400 | >400 | >400 | >400 | >400 | >400 |
| *Piperaceae* | | | | | | | | | | | | | | | |
| 68. | Cubeb EO | Berje Inc. | 290-148-4 | *Piper cubeba* L. | Singapore | >400 | 200 | 200 | 25 | >400 | >400 | 12.5 | 200 | 200 | >400 |
| 69. | Black pepper oleoresin 40/20 | Lluch Essence | 8002-56-0 | *Piper nigrum* L. | India | 100 | 800 | 800 | 800 | >400 | >400 | 400 | 800 | >400 | >400 |
| *Poaceae* | | | | | | | | | | | | | | | |
| 70. | Vetiver $CO_2$ | Ultra International | 8016-96-4 | *Chrysopogon zizanioides* (L.) Roberty syn. *Vetiveria zizanioides* (L.) Nash | India | >400 | 200 | 100 | 800 | 200 | 200 | 100 | 100 | 400 | 200 |
| *Ranunculaceae* | | | | | | | | | | | | | | | |
| 71. | Black seed expressed EO | Ultra International | 90064-32-7 | *Nigella sativa* L. | India | 25 | 800 | >400 | 800 | 200 | 200 | >400 | >400 | 200 | 200 |
| *Rosaceae* | | | | | | | | | | | | | | | |
| 72. | Rose absolute | Ultra International | 8007-01-0 | *Rosa x damascena* Mill. | Bulgaria | 400 | >400 | >400 | >400 | >400 | 400 | >400 | >400 | >400 | >400 |
| 73. | Rose absolute | Ultra International | 8007-01-0 | | Morocco | >400 | >400 | >400 | >400 | >400 | 400 | >400 | >400 | >400 | >400 |
| 74. | Rose EO | Ultra International | 8007-01-0 | | Bulgaria | 800 | >400 | >400 | >400 | >400 | >400 | >400 | >400 | >400 | >400 |
| 75. | Rose EO | Ultra International | 8007-01-0 | | Turkey | >800 | >400 | >400 | >400 | >400 | 400 | >400 | >400 | >400 | >400 |

| | | | | | | | | | | | | | | | |
|---|---|---|---|---|---|---|---|---|---|---|---|---|---|---|---|
| *Rubiaceae* | | | | | | | | | | | | | | | |
| 76. | Coffee Arabica $CO_2$ | Ultra International | 8001-67-0 | *Coffea arabica* L. | India | >400 | >400 | >400 | >400 | >400 | >400 | >400 | >400 | >400 | >400 |
| 77. | Coffee Robusta $CO_2$ | Ultra International | 8001-67-0 | *Coffea canephora* Pierre ex Froehner | India | >400 | >400 | 800 | >400 | >400 | 200 | >400 | >400 | >400 | >400 |
| 78. | Coffee EO | Berje Inc. | 8001-67-0 | *Coffea arabica* L. | El Salvador | 200 | >400 | 800 | 800 | >400 | 100 | 200 | >400 | >400 | >400 |
| *Rutaceae* | | | | | | | | | | | | | | | |
| 79. | Buchu leaf EO | Berje Inc. | 68650-46-4 | *Agathosma betulina* (P.J. Bergius) Pillans | South Africa | >400 | >400 | >400 | >400 | >400 | 200 | 400 | >400 | >400 | >400 |
| 80. | Buchu leaf EO | Berje Inc. | 68650-46-4 | *Agathosma crenulate* L. | South Africa | >400 | >400 | >400 | >400 | >400 | 200 | 400 | >400 | >400 | >400 |
| 81. | Buchu leaf EO | Ultra International | 68650-46-4 | *Agathosma betulina* Berg. | South Africa | >400 | >400 | >400 | >400 | >400 | >400 | >400 | >400 | >400 | >400 |
| 82. | Grapefruit red EO | Lebermuth | 8016-20-4 | *Citrus x paradisi* Macfad. | USA | 400 | >400 | >400 | >400 | >400 | >400 | >400 | >400 | >400 | >400 |
| 83. | Kumquat EO | Ultra International | N/A | *Fortunella japonica* Swingle | Brazil | >400 | >400 | >400 | >400 | >400 | >400 | >400 | >400 | >400 | >400 |
| 84. | Mandarin red EO | Berje Inc. | 8008-31-9 | *Citrus reticulata* Blanco syn. *Citrus nobilis* Andrews | Argentina | >400 | >400 | >400 | >400 | >400 | >400 | >400 | >400 | >400 | >400 |
| 85. | Orange bitter (fruit), distilled EO | A. Fakhry & Co. | 68916-04-1 | *Citrus* x *aurantium* L. var. *amara* | Egypt | 800 | >400 | >400 | >400 | >400 | >400 | 400 | >400 | >400 | >400 |
| 86. | Orange blood EO | Ultra International | 8008-57-9 | *Citrus sinensis* (L.) Osbeck | Italy | >400 | >400 | >400 | >400 | >400 | >400 | >400 | >400 | >400 | >400 |
| *Santalaceae* | | | | | | | | | | | | | | | |
| 87. | Australian sandalwood EO | Dutjahn Sandalwoods Oils | 8024-35-9 | *Santalum spicatum* (R.Br.) A. DC syn. *Eucarya spicata* (R.Br.) Sprag et Summ. | Australia | >400 | 400 | 100 | 400 | 800 | >400 | 200 | 400 | 800 | >400 |
| *Schisandraceae* | | | | | | | | | | | | | | | |
| 88. | Star anise, type $CO_2$ | Ultra International | 84650-59-9 | *Illicium verum* Hook. f. | India | >400 | >400 | >400 | 800 | >400 | >400 | 400 | >400 | >400 | >400 |
| 89. | Anise EO | Berje Inc. | 283-518-1 | *Illicium verum* Hook. f. | China | 400 | >400 | >400 | >400 | >400 | 200 | 400 | >400 | >400 | >400 |
| *Scrophulariaceae* | | | | | | | | | | | | | | | |
| 90. | Buddawood EO | Ultra International | 1429902-59-9 | *Eremophila mitchellii* Benth. | Australia | >400 | 800 | 800 | >400 | >400 | >400 | 400 | 800 | 800 | >400 |
| *Solanaceae* | | | | | | | | | | | | | | | |
| 91. | Capsicum oleoresin 1.000.000 SHU | Lluch Essence | 8023-77-6 | *Capsicum annuum* var. *annuum* L. | India | 400 | >400 | >400 | >400 | >400 | >400 | >400 | >400 | >400 | >400 |
| 92. | Paprika Oleoresin 40.000 CU | Lluch Essence | 84625-29-6 | *Capsicum annuum* L. | India | >400 | >400 | >400 | >400 | >400 | >400 | >400 | >400 | >400 | >400 |
| 93. | Paprika Oleoresin 80.000 CU | Lluch Essence | 84625-29-6 | | India | >400 | >400 | >400 | >400 | >400 | >400 | >400 | >400 | >400 | >400 |
| *Valerianaceae* | | | | | | | | | | | | | | | |
| 94. | Valerian EO | Berje Inc. | 616-930-0 | *Valeriana officinalis* L. | China | >400 | >400 | >400 | >400 | >400 | 400 | >400 | >400 | >400 | >400 |
| *Verbenaceae* | | | | | | | | | | | | | | | |
| 95. | Verbena EO | Lluch Essence | 8024-12-2 | *Lippia citriodora* Kunth. | Morocco | >400 | >400 | >400 | >400 | >400 | >400 | 200 | >400 | >400 | >400 |
| *Vitaceae* | | | | | | | | | | | | | | | |
| 96. | Cognac white EO | Berje Inc. | 232-403-4 | *Vitis vinifera* L. | USA | 100 | 800 | >400 | 800 | >400 | 100 | 200 | >400 | 800 | >400 |
| | *Zingiberaceae* | | | | | | | | | | | | | | |
| 97. | Cardamom green $CO_2$ | Ultra International | 8000-66-6 | *Elettaria cardamomum* (L.) Maton | India | 400 | 800 | >400 | >400 | >400 | >400 | 400 | 800 | 800 | >400 |
| 98. | Ginger EO | Ultra International | 8007-08-7 | *Zingiber officinale* Roscoe | India | >400 | 200 | 100 | 400 | >400 | 100 | >400 | 800 | 800 | >400 |
| 99. | Turmeric $CO_2$ | Ultra International | 8024-37-1 | *Curcuma longa* L. | India | >400 | >400 | >400 | >400 | >400 | >400 | >400 | >400 | >400 | >400 |
| | Antibiotics [µg/ml] | | | | | | | | | | | | | | |
| | Gentamicin | | | | | 5.000 | 1.000 | | | | 2.000 | 0.625 | | | |
| | Ciprofloxacin | | | | | | 0.625 | | | | 0.008 | 0.019 | 0.250 | 0.019 | 0.500 |
| | Ampicillin | | | | | | | 0.0625 | 0.250 | 0.008 | | | | | |
